# A Fully-Integrated Ambient RF Energy Harvesting System with 423-μW Output Power

**DOI:** 10.3390/s22124415

**Published:** 2022-06-10

**Authors:** Kishore Kumar Pakkirisami Churchill, Harikrishnan Ramiah, Gabriel Chong, Yong Chen, Pui-In Mak, Rui P. Martins

**Affiliations:** 1 Department of Electrical Engineering, Faculty of Engineering, University of Malaya, Kuala Lumpur 50603, Malaysia; kva180030@siswa.um.edu.my; 2 Centre of Research Industry 4.0 (CRI 4.0), Faculty of Engineering, University of Malaya, Kuala Lumpur 50603, Malaysia; 3 Nexperia R&D Penang, Bayan Lepas 11900, Malaysia; gabriel.chong@nexperia.com; 4 State-Key Laboratory of Analog and Mixed-Signal VLSI/IME, Department of Electrical and Computer Engineering, Faculty of Science and Technology, University of Macau, Macau 999078, China; ychen@um.edu.mo (Y.C.); pimak@um.edu.mo (P.-I.M.); rmartins@um.edu.mo (R.P.M.); 5 Instituto Superior Técnico, Universidade de Lisboa, 1099-085 Lisboa, Portugal

**Keywords:** RF energy harvesting (RFEH), CMOS, RF-DC, DC-DC, charge pump, clock generator, ring-VCO, shared-dynamic-biasing

## Abstract

This paper proposes a 2.4-GHz fully-integrated single-frequency multi-channel RF energy harvesting (RFEH) system with increased harvested power density. The RFEH can produce an output power of ~423-μW in harvesting ambient RF energy. The front-end consists of an on-chip impedance matching network with a stacked rectifier concurrently matched to a 50 Ω input source. The circuit mitigates the “dead-zone” by enhancing the pumping efficiency, achieved through the increase of V_gs_ drivability of the proposed internal gate boosting 6-stage low-input voltage charge pump and the 5-stage shared-auxiliary-biasing ring-voltage-controlled-oscillator (VCO) integrated to improve the start-up. The RFEH system, simulated in 180-nm complementary metal–oxide–semiconductor (CMOS), occupies an active area of 1.02 mm^2^. Post-layout simulations show a peak power conversion efficiency(PCE) of 21.15%, driving a 3.3-kΩ load at an input power of 0 dBm and sensitivity of −14.1 dBm corresponding to an output voltage, V_out,RFEH_ of 1.25 V.

## 1. Introduction

Ubiquitous wireless sensor nodes (WSNs), body sensor nodes (BSN), and portable health monitoring devices will require energy harvesting (EH) to achieve battery-less or long-term battery-assist operation. RF Energy Harvesting (RFEH) [1,2,3,4,5,6,7] is gaining interest due to its continuous availability in urban environments compared to other renewable energy sources like solar, thermal, and piezo EH. In an indoor environment, the RF signal is concentrated, and in the outdoor environment the weather, season, and illumination have less impact around 0.0001 to 0.004 dB/m as propagation attenuation of EM waves [2]. In addition, the RFEH system conciliates well with existing wireless systems with a standard 50-Ω impedance matching network, requiring only an antenna as a transducer [8]. Most modern communication devices enable Wi-Fi or Bluetooth low-energy (BLE), hence, RF energy is abundant and the 2.4-GHz frequency band is of interest to achieve a small physical form factor as well as a low-cost solution for RFEH [8]. According to the International Telecommunication Union (ITU), global Wi-Fi growth increased by 80% after the first quarter of 2020 [9]. Hence, the power density of the wireless frequency in this channel [10] increased and we can drain it to overcome the limitation due to the low input power density of the RFEH in improving the system’s energy reliability. In complementing the RFEH, the power consumption of various portable devices operates within the hundreds of µW range for the detection of symptomatic patterns in audio biological signals [11], ECG [12,13], and other health monitoring [14]. Table 1 shows the typical range of the power consumption of various electronic devices. 

The usage of multi-channel RFEHS not only has the potential to improve the performance of the harvester but also increases the harvesting sensitivity [17], and supports a multiple-input multiple-output (MIMO) system [18,19].

**Figure 1:**
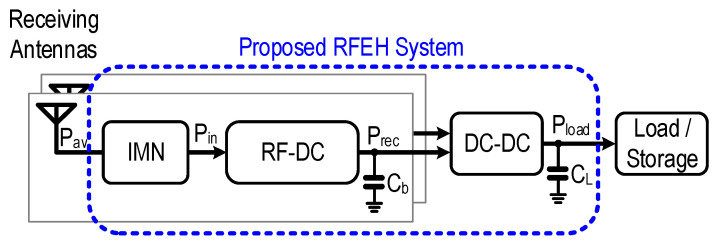
Single-frequency multi-channel RF energy harvesting system.

Various multi-channel, multi-band RFEH systems adopting discrete elements require off-chip components [1,4,10,17,18,19,20], which include Schottky diodes [15] and rectenna [21]. Figure 1 presents the simple block diagram of the proposed RFEH system, which includes an on-chip impedance matching network (IMN), a stacked rectifier, and a charge-pump-based DC-to-DC converter with the developed shared-auxiliary-biasing ring-voltage-controlled-oscillator (SAB-RVCO). The array of antennas receives the available RF energy from the free space environment. The rectifier converts the time-varying electromagnetic RF signal to a DC voltage. The impedance matching network (IMN) interfaces the antenna (typically 50 Ω) and rectifiers to reduce impedance mismatch which in turn minimizes the power reflection. However, the physical form factor of the discrete components occupies a huge space, this limits the EH in the targeted WSN, BSN, and health care application. To overcome the aforementioned challenges, recent research directions changed towards solutions on-chip or with full integration. The primary scope of the proposed work is to increase the higher power density with higher power conversion efficiency and higher sensitivity for an on-chip solution. 

Challenges of the on-chip IMN solution: IMN in RFEHS is built with capacitors and high Q-inductors for voltage amplification and maximum power transfer [22,23,24,25,26,27,28]. However, designing a high Q-inductor in complementary metal–oxide–semiconductor (CMOS) technology has its limitations and trade-offs in attaining maximum power conversion efficiency [29]. 

To overcome the aforementioned challenges of an on-chip IMN design, we present the following findings: (1) Through the investigation of the on-chip impedance matching technique for the RFEH system [30,31], this work proposes a design strategy that adopts the variation of the rectifier input impedance and devises a technique for the area reduction along with performance enhancement. (2) To increase the sensitivity of the RFEH system, we also put forward a low-power, non-overlap-clock shared-auxiliary-biasing ring-voltage-controlled-oscillator (SAB-RVCO) and the proposed internal gate biasing charge pump. By using a novel body-biasing technique, the SAB-RVCO can generate a non-overlap clock at low supply headroom and improve the pumping efficiency of the charge pump. The design of the proposed SAB-*R*VCO uses a dual-input RFEH system achieving single-chip integration and an overall low-power consumption for the harvester.

This paper reports a dual-channel fully-integrated RFEH system that consumes a total chip area of 1.02-mm^2^ in 180-nm CMOS. With the proposed dual-channel scheme, the circuit exhibits a higher power density of 423 μW at 0-dBm input power. Section 2 describes the overview of the proposed fully-integrated RFEH system. Section 3 presents the IMN design strategy and circuit-level design of the SAB-RVCO along with the charge-pump circuits. We also discuss the control circuit adopted in the RFEHS. Section 4 highlights the post-layout simulation results of the RFEHS and Section 5 concludes the work. 

## 2. System Architecture

**Figure 2:**
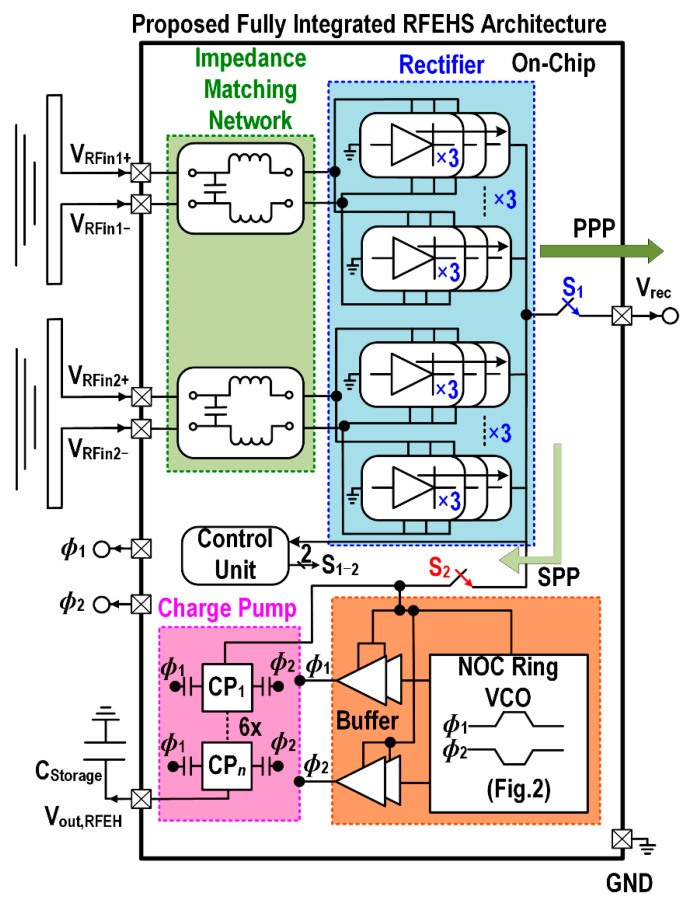
Block diagram of fully-integrated dual-input RF energy harvesting (RFEH) system.

Figure 2 outlines the conceptual block diagram of a fully-integrated dual-input RFEH system. Two differential antennas configured at the same frequency will scavenge RF power to increase the harvesting input density. The integrated rectifier has a highly-efficient differential drive similar to [32]. The proposed rectifier is cascaded in a 3 × 3 stage to achieve high efficiency and low input impedance. An on-chip differential LC-matching is adopted and realized in a fully-integrated system. 

The proposed system consists of two power paths controlled by the control unit incorporating two MOSFET switches, an inverter, and a common-gate input comparator. Figure 2 shows the primary power path (PPP) as well as the secondary power path (SPP) which harvests high power directly to the load unit and low power to the storage unit in extending the lifetime of the battery. The comparator weighs the rectifier output voltage (V_rec_) and the reference voltage (V_ref_). As long as V_rec_ is greater than V_ref_, the comparator output voltage V_cmp_ is low and the inverter output will be high.

This activates the PPP in which the control unit selects switch S_1_ (HIGH) to turn on and S_2_ (LOW) to turn off. This in turn increases the PCE of the RFEH system during the high power mode. Similarly, if the comparator weighs in V_rec_ to be lower than the V_ref_, the voltage V_cmp_ becomes high and inverter output is low, hence the control unit switches S_1_ to turn-off and S_2_ to turn-on. This operation activates the SPP of the RFEHS. In short, this mode benefits in increasing the sensitivity of the RFEH system through the proposed charge pump. 

## 3. Circuit Details

### 3.1. Rectifier

**Figure 3:**
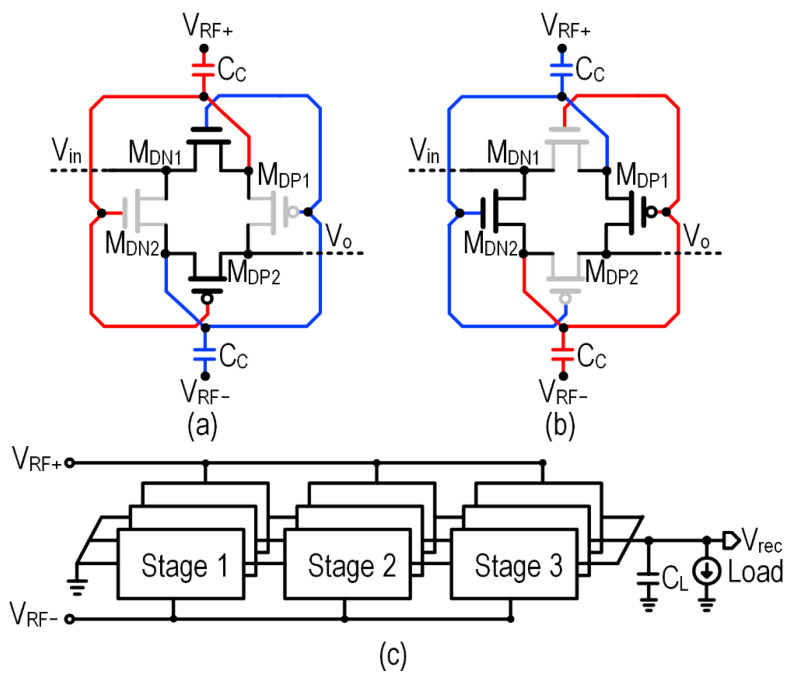
RFEH front-end: (**a**,**b**) Operation of differential cross-coupled (DCC) rectifier and (**c**) Proposed 3 × 3 rectifier.

The proposed RFEH system uses a customized differential cross-coupled (DCC) [33] rectifier with three-stage series with three parallel stages to achieve impedance transformation, which is discussed in detail in Section 3.2. The proposed rectifier attains higher PCE at low input power compared to the conventional diode-connected single-ended rectifier topology. The rectifier is configured with the transistors M_DP1_, M_DP2_, M_DN1_, and M_DN2_ which are actively biased with differential signals V_RF+_ and V_RF-_ as shown in Figure 3. When V_RF+_ is negative and V_RF-_ is in a positive cycle, the transistor M_DN1_ is forward biased due to the positive gate voltage from V_RF-_ which reduces the on-resistance of M_DN1_. Alternately, when V_RF+_ is positive and V_RF-_ is in a negative cycle, M_DN1_ is reverse biased by a decrease in the gate voltage, and thus reduces the reverse leakage current. Previously-reported work on the fully-integrated RFEHS adopts a single-ended rectifier due to the size constraint of the on-chip inductor. The proposed RFEH system in this work is the first to couple the DCC rectifier with an on-chip matching network. In order to reduce the switching losses, an optimal number of fixed rectifier stages are used instead of reconfigurable stages. 

### 3.2. Impedance Matching Network 

**Figure 4:**
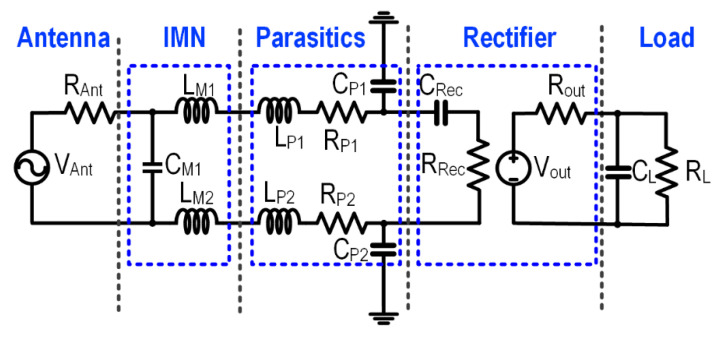
Equivalent circuit of the RFEH front-end.

The design of the IMN network aims to match the rectifier to a standard 50 Ω source impedance. The equivalent model of the IMN circuit is shown in Figure 4. The voltage source with an amplitude of V_Ant_ is an integrated series with the antenna resistance R_Ant_, and with an IMN that consists of inductor L_M1_, L_M2_, and capacitor C_M1_. In addition to impedance transformation to attain maximum power transfer, the IMN is also utilized for passive voltage amplification.

The proposed IMN network introduces an impedance reduction technique, which consequently improves the Q-factor of the inductor. The realization of the IMN is evaluated through the input reflection coefficient, S_11_, which is defined as,

(1) $$S_{11}\;\left(\mathrm{dB}\right)=\Gamma =\frac{Z_{\mathrm{Rec}}-Z_{\mathrm{Ant}}^{*}}{Z_{\mathrm{Rec}}+Z_{\mathrm{Ant}}^{*}}$$

where Z_Rect_ = R_Rec_ + X_Rec_ is the impedance of the rectifier and $Z_{\mathrm{Ant}}^{*}$ is the complex conjugate of Z_Ant_, where Z_Ant_ = R_Ant_ + X_Ant_ is the impedance of the antenna or source. The amount of power reflected is calculated through Equation (2),

(2) $${\left|\Gamma \right|}^{2}={\left|\frac{Z_{\mathrm{Rec}}-Z_{\mathrm{Ant}}^{*}}{Z_{\mathrm{Rec}}+Z_{\mathrm{Ant}}^{*}}\right|}^{2}$$

The passive voltage gain A_v_ is directly proportional to the Q_L_-factor of the inductor [30],

(3) $$A_{V}=\frac{1}{2}\;\sqrt{1+Q_{L}^{2}}$$

Based on Equation (3), Q_L_ is a defining factor for voltage gain, which is directly proportional to the PCE of the RFEH system. At the operating frequency of 2.4 GHz in the 180-nm CMOS platform, the inductor quality (Q) factor is in the range of 8 to 1 for 6 nH to 22 nH of inductance. The Q-factor is limited beyond the defined range of inductance with the silicon chip area increasing proportionally with the inductor value. The conventional IMN design strategy is based on the rectifier’s optimal number of stages, hence the rectifier input impedance is fixed. The balanced or symmetry impedance matching is used to derive the circuit with the same impedance and effect of the signal traveling in both directions of the port.

**Figure 5:**
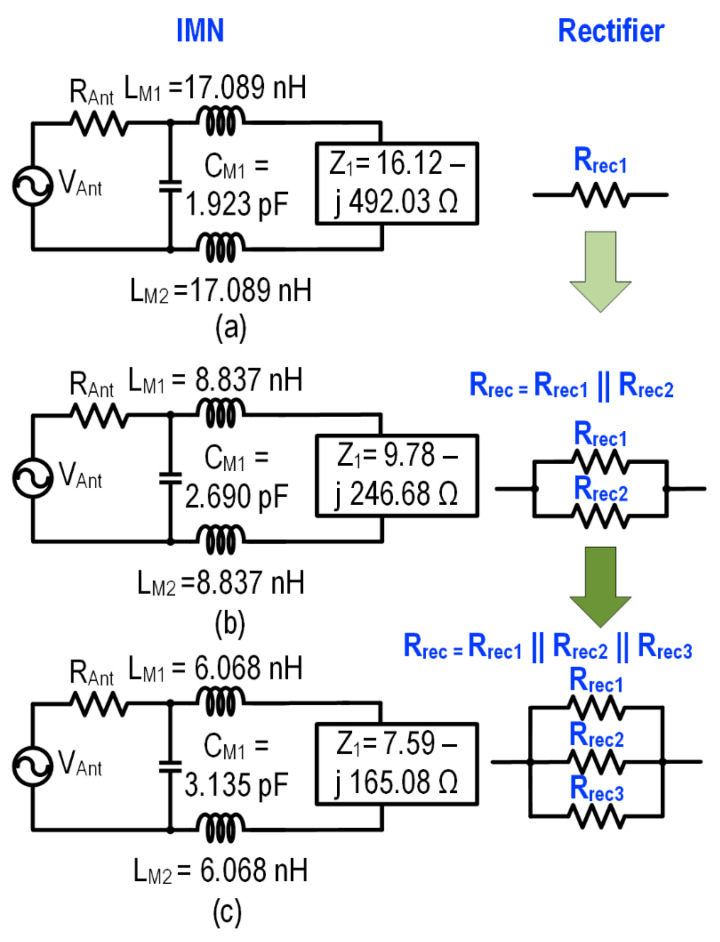
Impedance reduction strategy of RF front-end: (**a**) 3-stage rectifier, (**b**) 2 parallel—3-stage rectifier, and (**c**) 3 parallel—3-stage rectifier.

An on-chip LC-match IMN is adopted as shown in Figure 5. The value of C_m_ and L_m_ can be approximated by [34],

(4) $${\;C}_{m}=\frac{1}{\omega_{\mathrm{RF}}Z_{\mathrm{Rec}}}\sqrt{\frac{Z_{\mathrm{Rec}}}{Z_{\mathrm{Ant}}}-1}$$

(5) $$L_{m}=\frac{1}{\omega_{\mathrm{RF}}^{2}C_{m}}$$

where ωRF represents the input RF frequency. The ideal simulated input impedance of the 3-stage series rectifier is 16.12 − j492.03 Ω, with the respective on-chip IMN component value at 2.4 GHz calculated as L_M1_ = L_M2_ = 17.089 nH at a Q of 3.15, and the capacitance C_M1_ = 1.923 pF. The computed component value corroborates with Equation (3) and reduces A_v_ and the PCE of the system. The proposed system aims to improve the voltage gain by increasing the Q of the inductor. In the proposed RFEHS, a 3-stage series rectifier is connected in parallel to reduce rectifier impedance, thus reducing inductance and improving the Q factor as well as the passive voltage gain which concurrently improves the PCE and achieves a smaller silicon area. Figure 5 shows the impedance reduction scheme with a single 3-stage series rectifier, 2-parallel-3-stage series rectifier, and 3-parallel-3-stage series rectifier. The input impedance of the rectifier with 2 and 3 parallel stages is 9.78 − j246.68 Ω and 7.59 − j165.08 Ω, respectively. This reduction of impedance improves the inductor Q value to ~6 for two parallel stage rectifiers and 8 for 3-parallel stage rectifiers in the technology of choice. Similarly, a two-channel symmetrical system is designed with an on-chip impedance matching network.

### 3.3. Proposed SAB-RVCO

A step-up DC-DC converter is an essential unit in attaining high sensitivity for RFEHS. The capacitive-based step-up DC-DC converter or charge pump (CP) is best suited for an on-chip implementation in contrary to the inductor-based DC-DC converter which requires bulky off-chip components which is unfavorable for miniaturization of the device. The monolithic ring-voltage control oscillator (R-VCO) is the main peripheral of the charge pump, where low-power and low start-up will be the key performance parameter for the RFEHS [30].

The primary bottleneck in designing a charge pump for the EH system is to achieve a low-power, low-startup RVCO as well as a non-overlap clock unit. In addition, the RVCO must be able to operate with a minimum supply voltage (V_DD_,_min_), lower than the threshold voltage (V_th_) of the transistor. These limitations are dependent on the subthreshold swing (S_S_) of the CMOS process. The range of Ss is between 70 to 100 mV/decade with the advanced CMOS process node can achieve nearer to the Meindl limits [35]. The oscillation of RVCO must exhibit a rail-to-rail output with higher current drivability to drive the pumping capacitor of the CP. However, when V_rec_ is less than V_th_, the device limits the current flow due to weak inversion between source and drain channel, reducing the RVCO drivability. In a higher process node technology, the V_th_ of the device decreases, however, this, in turn, increases the production cost. Alternatively, the body bias technique favorably reduces the V_th_ and increases the device inversion region in lower process technology. 

The V_th_ of an N-channel metal-oxide-semiconductor (NMOS) is expressed as [36],

(6) $$V_{\mathrm{th}}=V_{t0}+\gamma \left(\sqrt{2\left|\phi_{F}\right|+V_{\mathrm{sb}}}-\sqrt{2\left|\phi_{F}\right|}\;\right)$$

where γ is the body-effect coefficient, ϕ_F_ is the Fermi potential, V_th_ and V_th0_ are the threshold voltage and threshold voltage at zero source-to-bulk voltage (V_sb_), respectively. 

The deep-well technology enables the body bias technique which varies the V_th_ and allows a low voltage headroom operation. To minimize the V_th_ of the transistor, the V_sb_ is sourced with a higher voltage than the V_ss_ for NMOS and alternately sourced with a lower voltage than the V_dd_ for a P-channel metal-oxide-semiconductor (PMOS) device. This reduces the depletion region and increases the near saturation region operation, which in turn increases the saturation current and concurrently reduces the propagation delay. The commonly used body biasing technique includes the dynamic threshold-voltage MOSFET (DTMOS) [37], swapped body bias (SBB) [38], and auxiliary transistor [39]. In the DTMOS configuration, the gate terminal is connected to the body or bulk of the PMOS and is similar to an NMOS transistor. Hence, the V_th_ of the transistor changes dynamically concerning the gate-source voltage (V_gs_) of the inverter. Assuming a single-stage inverter with DTMOS applied between the supply voltage (V_dd_) and ground, if V_gs_ is low, then the bulk of both PMOS and NMOS should be low. Hence, V_bs_ of the PMOS and NMOS are driven to forward bias (V_bsp_ = −V_dd_) and zero-bias (V_bsn_ = 0), respectively. If V_gs_ = V_dd_, then the bulk of both the PMOS and NMOS should be high, hence V_bs_ of the PMOS and NMOS are driven to zero-bias (V_bsp_ = 0) and forward bias (V_bsn_ = V_dd_), respectively. In the SBB configuration, the bulk of the PMOS are connected to ground whereas the bulk of the NMOS transistors are connected to V_dd_, so that V_bs_ of both the PMOS and NMOS are driven to forward bias with V_bsp_= −V_dd_ and V_bsn_ = V_dd_, respectively.

In an auxiliary transistor body biasing configuration, the bulk of the PMOS and NMOS transistor is connected with the drain voltage of the auxiliary NMOS and PMOS, respectively. This achieves a higher biasing voltage magnitude compared to other body biasing techniques. However, the additional transistor increases the size as well as the effect of the inherent parasitic, which causes a mismatch in the phase shifters and tends to degrade the CP pumping efficiency at low voltage [40]. 

**Figure 6:**
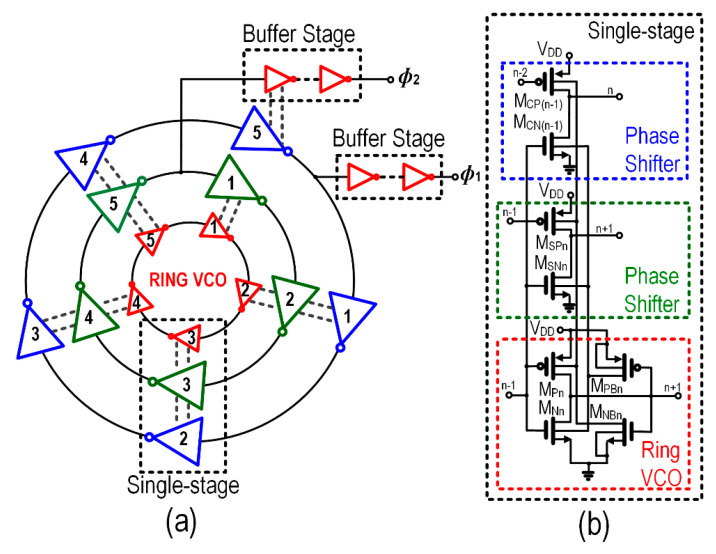
Details of the proposed 5 stages (N = 1, 2,…, 5) *R*VCO: (**a**) *R*VCO for energy harvesting, and (**b**) Shared-auxiliary-biasing ring-voltage-controlled-oscillator (SAB-*R*VCO).

The architecture of the proposed SAB-RVCO is shown in Figure 6a. With n representing the stage number, the core circuitry of the RVCO shown in Figure 6b consists of the transistor M_Pn_ and M_Nn_, two-phase shifters comprising of M_SPn_, M_SNn_, M_CPn_, and M_CNn_, which generate two-phase clocks *Φ*_1_ *Φ*_1_ and *Φ*_2_. In the transistor’s weak-inversion region, the parasitic dominates, and the clock signals (*Φ*_1_ and *Φ*_2_) overlap, which degrades the CP performance. To overcome this problem, transistors M_PBn_ and M_NBn_ are integrated to establish bulk-biasing for the RVCO and phase shifters. The bulk-biasing through M_PBn_ and M_NBn_ ensures *Φ*_1_ and *Φ*_2_ are non-overlap clock signals which improve the pumping efficiency of the charge pump when V_DD_ is low. The minimum size of the device is maintained to achieve lower V_th_ in RVCO while the dimension of the buffer is maximized by a systematic transistor sizing strategy [41] to drive the CP pumping capacitance approximating to 60 pF as well to minimize the rise-time and fall-time edge of the clock generation.

**Figure 7:**
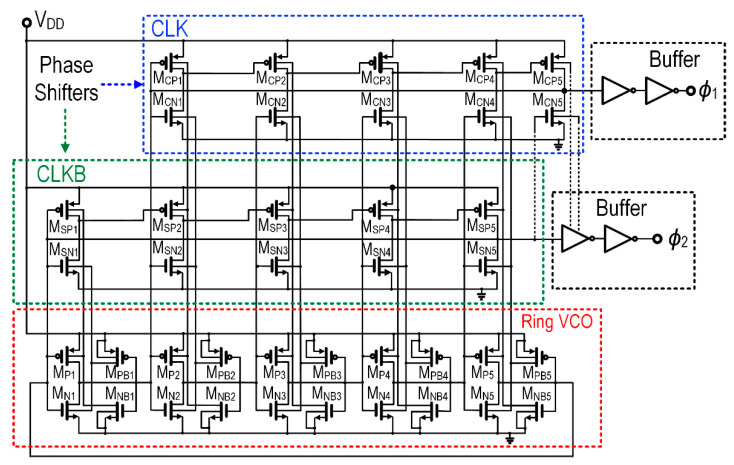
Complete schematic of proposed low-start-up CMOS SAB-*R*VCO.

**Figure 8:**
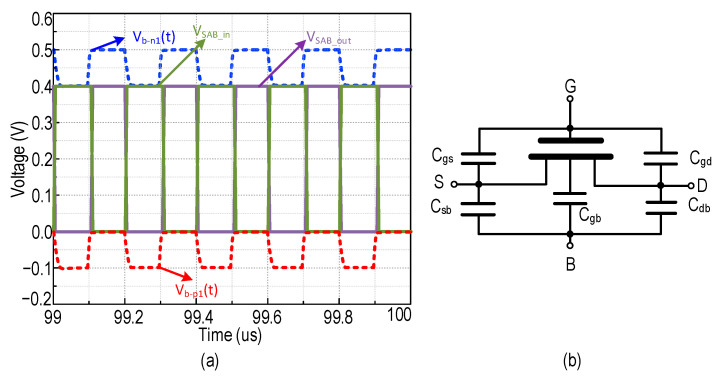
(**a**) Simulated waveform of the output voltage (V_OUT_) of the single-stage SAB-RVCO scheme (**b**) MOSFET parasitic capacitances.

Figure 7 shows the schematic of the SAB-RVCO with two parallel phase shifter circuits. The number of stages (N) of the RVCO is attained based on the study of an effective number of stages relations with the frequency (f_Osc_) [42], power consumption (P_c_) [43], and losses of CP [30]. The oscillation of frequency and power is given by [42,43],

(7) $$f_{\mathrm{Osc}}=\frac{1}{N\cdot 2\cdot t_{d}}\;$$

(8) $$P_{C}={\mathrm{Nf}}_{\mathrm{Osc}}C_{L}V_{\mathrm{DD}}^{2}$$

where the N is inversely proportional to the frequency and directly proportional to the power consumption [42]. It is worth noting that the frequency is directly proportional to the power consumption. Based on the inference from [30], higher frequency causes switching losses in the CP circuit. It is worth noting that switching losses are inversely proportional to the conduction loss. Hence the optimal 5-stages of the proposed RVCO achieve lower startup and higher efficiency, with lower parasitics effect, where N is the odd number of inverters (N = 5). The oscillation frequency of the RVCO is dependent on the time delay of each inverter stage, where the operation is shown in Figure 8a. Referring to Figure 7, in the SAB-RVCO, the bulk terminal of M_P1_ and M_N1_ are biased by the drain voltage of the auxiliary transistor M_NB1_ and M_PB1_, respectively. M_NB1_ provides the initial voltage for the parasitic capacitance of the transistor M_P1_ (C_db-p1_, C_sb-p1_) and M_NB1_ (C_gd-nb1_, C_db-nb1_), where the parasitics of MOSFET are descriptively shown in Figure 8b. When V_g_ of M_P1_ is low, the drain voltage of M_NB1_ pulls V_b-p1(t)_ to the ground. This satisfies the initial condition of the bulk of M_P1_.

(9) $$V_{b-p1\;}\left(0\right)={\;V}_{d}\;\left(0\right)-{\;V}_{\mathrm{dd}}\;$$

When V_d(t)_ falls too low, V_b-p1(t)_ reduces below zero and reaches a negative value due to the discharge of capacitance C_db-p1_ and C_gd-nb1_. Alternately, when V_d(t)_ reaches a high value, then V_b-p1_ increases from a negative voltage to zero due to the charging of capacitance C_db-p1_ and C_gd-nb1_. During the process of charging and discharging the internal capacitance, V_b-p1(t)_ is lesser than the source voltage of M_NB1_, and no current flows between drain to source of M_NB1_, hence, M_NB1_ acts as capacitor C_gd-nb1_. M_PB1_ provides the initial voltage for the parasitic capacitance of the transistor M_N1_ (C_db-n1_, C_sb-n1_) and M_PB1_ (C_gd-pb1_,C_db-pb1_). When V_g_ of M_N1_ is high, the drain voltage of M_PB1_ drives V_b-n1(t)_ to V_dd_. This satisfies the initial condition of the bulk of *M*_N1_.

(10) $$V_{b-n1}\;\left(0\right)={\;V}_{d}\;\left(0\right)+{\;V}_{\mathrm{dd}}$$

When V_d(t)_ is high, V_b-n1(t)_ increases above V_dd_ and reaches a positive value due to the charging of C_db-n1_ and C_gd-pb1_. Alternatively, when V_d(t)_ reaches a low value, V_b-n1_ reduces from a higher positive value to V_dd_ due to discharging of capacitor C_db-n1_ and C_gd-pb1_. During the process of charging and discharging the internal capacitance, V_b-n1(t)_ is higher than the source voltage of M_PB1_ and there is no current flow between drain to source of M_PB1_, hence the transistor M_PB1_ acts as capacitor C_gd-pb1_.

The auxiliary transistor acts as a capacitor during the charging and discharging phase that provides |V_bs-n1_| ≥ V_dd_ and |V_bs-p1_| ≤ 0 compared to DTMOS and SBB, then RVCO operates in the sub-threshold region, the transistor current has an exponential relation with V_bs_ and a linear relation concerning the aspect ratio. By reducing the aspect ratio, the subsequent stage of the transistor’s internal capacitance (C_o_) should be smaller, which requires less drain to the source current which assists to maintain low power dissipation. 

The auxiliary transistor biasing technique shows improvement in the performance of the RVCO, phase shifter, and buffer circuit. However, the design technique doubles the area by the ratio of 1:1 (Primary transistor: Auxiliary transistor), increasing the parasitics and leading to mismatch at the phase of the clock which is unfavorable for low voltage CP below 250 mV [40,44]. To reduce the inherent parasite and improve the area efficiency of the clock generation unit, a single set of the auxiliary biasing transistors is shared between the parallel stage of inverters which operates with similar logic behavior in a configuration as the 3rd and 2nd stages of the phase shifter cell are connected with the 3rd stage of the RVCO inverter cell. Through this vertical sharing bias technique, our work achieves an area reduction in a ratio of 3:1 without compromising the functionality. In addition, the proposed SAB-RVCO achieves a similar drain to source current with lower input voltage due to the higher biasing voltage magnitude.

### 3.4. Charge-Pump Realization

The capacitive charge pump (CP) is well adapted for monolithic implementation compared to the bulky inductor-based boost converter. In addition, the capacitive CP has a good driving capability, minimal parasitic effect, and low complexity well fitted for energy harvesting applications. The CP performance at low voltage relies on the charge transfer switching resistance, particularly in the Dickson CP operation. The NMOS is driven in the triode region, and for a latched CP configuration, both the NMOS and PMOS transistor are driven in the triode region. For simplicity of the expression, the on-resistance (R_on_) of the NMOS transistor is given neglecting the short channel effect,

(11) $$R_{\mathrm{on}}=\frac{1}{{µC}_{\mathrm{ox}}\frac{W}{L\;}\left(V_{\mathrm{gs}}-V_{\mathrm{th}}\right)}\;$$

where µ is the mobility of the electron/holes and C_ox_ is the oxide capacitance which is a technology-dependent parameter. The CP resistance can be reduced by increasing the gate-to-source voltage (V_gs_). The internal gate enhances overdrive voltage [45] and external clock boosting topology can provide 2 folds [46,47] or 3 folds [48] of input voltage for low voltage CP application. However, in most cases, the clock signal is equal to V_dd_, therefore, increasing the V_gs_ to greater than the V_dd_ is a tangible solution observed from Equation (9). The proposed CP provides clock amplitude equal to 2V_gs_ and achieves the primary goal of a CP circuit. 

**Figure 9:**
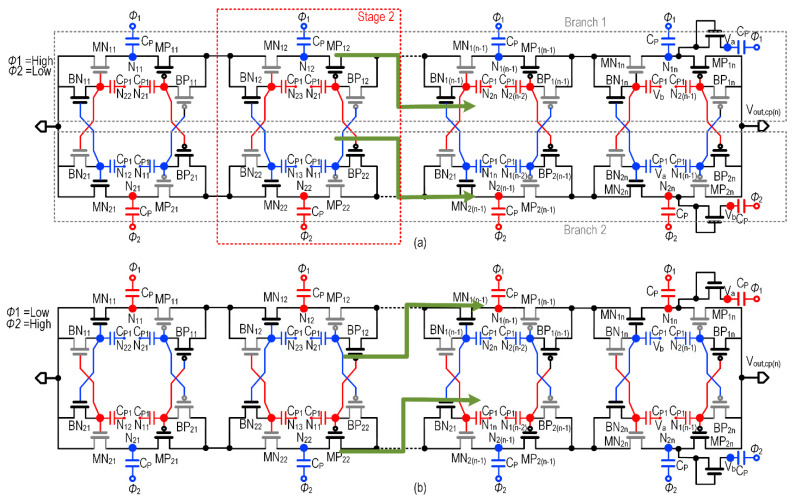
Schematic of the proposed charge pump: (**a**) operation in Cycle 1 (**b**) operation in Cycle 2.

Figure 9 shows the proposed 6-stage CP with the operation of each cycle described as follows. Each stage of the proposed CP consists of PMOS (M_P1i_, M_P2i_, B_P1i_, B_P2i_), NMOS (M_N1i_, M_N2i_, B_N1i_, B_N2i_), pumping capacitor (CP), and the boosting capacitor or (C_p1_) where “i” represents the number of stages (i = 1,2,…n).

The bulk terminal of both the PMOS and NMOS transistors are connected to the respective source terminal to eliminate the body effect. The clock signals (*Φ*_1,_ and *Φ*_2_) which have a clock amplitude up to V_dd_, are applied to the complementary CP pumping capacitors of branch 1 (B_1_) and branch 2 (B_2_). These branches are connected in parallel to the supply and output nodes. In response to *Φ*_1_ and *Φ*_2_ signals, the pumping capacitors in B_1_ and B_2_ charge alternately with V_dd,_ in a repetitive process as the charges are stored in the capacitors and transferred to the output V_OUT,CP_. 

The gate voltages of (MN_12_, BN_22_), (MN_22_, BN_12_), (MP_12_, BP_22_), and (MP_22_, BP_12_) are connected to the boosting capacitor through the nodes N_23_, N_13_, N_21_, and N_11_, respectively. In stage 2, the NMOS transistors are connected with the output node N_23_, N_13_ of the next immediate stage of the CP, and PMOS transistors are connected with the output node N_21_, N_11_ of the previous stage CP. This pattern of connection continues to the (*n* − 1)th stage. In the first stage, the gate voltage of the PMOS transistor is connected to the clock from the output node N_21_, N_11_ complimentary branches, due to the absence of a former stage. Besides, the *n*th stage has an additional pair of transistors with minimally sized capacitance to provide gate voltage for the *n*th stage NMOS transistor. To reduce leakage in the higher stage, the last three stages of the CP is constructed with thick oxide transistors, and the first three stages adopt nominal transistor for better conduction. 

**Figure 10:**
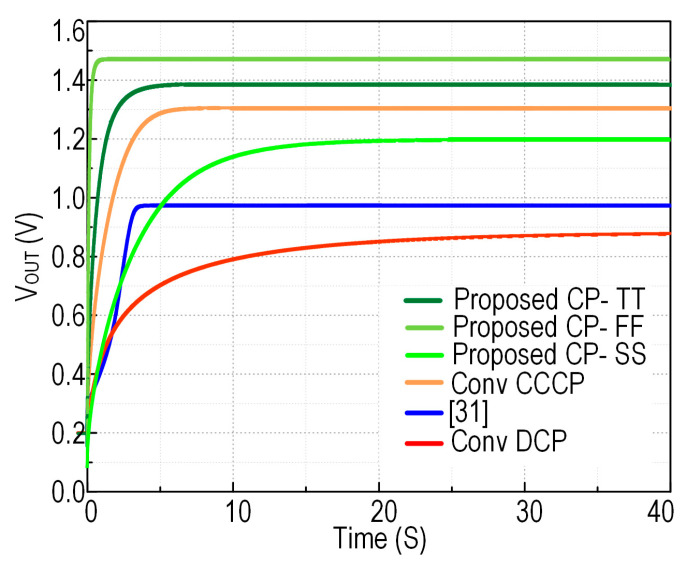
The transient output voltage of the proposed charge pump with prior art at V_dd_ = 200 mV.

A performance comparison with the prior art is shown in Figure 10 where the charge pump output voltage is plotted versus time. However, to justify the performance, the conventional cross-coupled charge pump (CCCP) and the transistor-based Dickson charge pump (DCP) are recreated and simulated under the same test conditions by using similar transistor sizing, pumping capacitance, and other design parameters. It is evident that the proposed solution achieves the highest performance in the adopted lower technology node.

### 3.5. Control Circuit 

**Figure 11:**
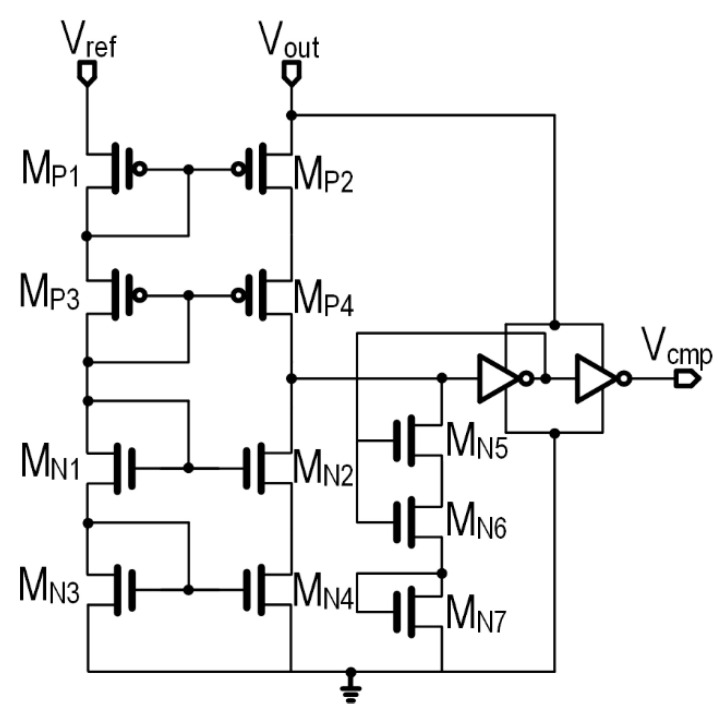
Common-gate comparator.

The control circuit design is mandatory in the proposed architecture due to the configuration of two power paths. S_1_ and S_2_ are controlled by the common-gate comparator [49,50] and an inverter where the comparator is the main block in the control unit, which is shown in Figure 11. Yet, under low power mode, the device works in the sub-threshold region, where the current has an exponential relation respective to V_ref_, indicating that it is negligible. As the adopted 180-nm CMOS is enabled via the long-channel device, this feature favorably results in a lower current consumption deviating from the low-current conduction of the transistor. 

## 4. Post-Layout Simulation Results and Comparison

**Figure 12:**
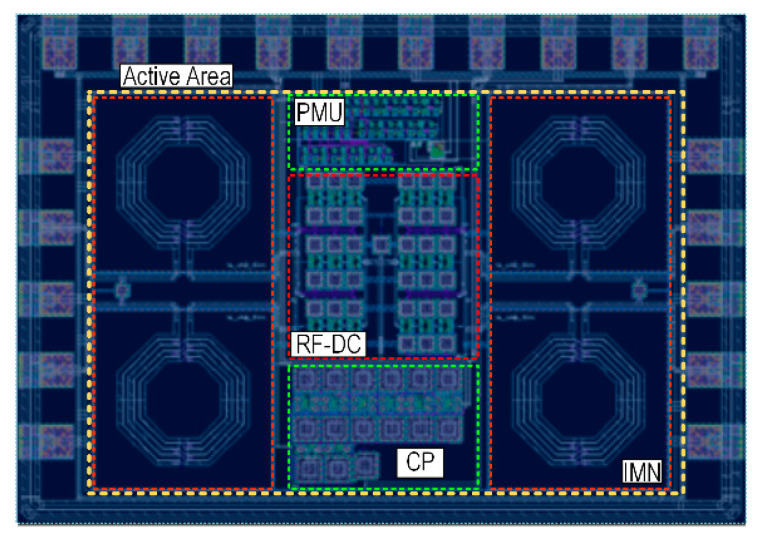
Chip Layout of the proposed RFEH System.

The proposed stack rectifier technique implemented along with the SAB-*R*VCO and charge pump is adopted in a fully integrated dual-input RF energy harvesting system and implemented in a 180-nm CMOS process shown in Figure 12, where the post-layout simulation result includes parasitic extraction of the parasitic capacitance, parasitic resistance, and parasitic inductance to create an accurate model of the circuit. Table 2 shows the transistor sizing of the proposed system. The active chip area of the entire system is 1.02 mm^2^ while the proposed SAB-*R*VCO consumes an active silicon area of 0.037 mm^2^.

The PCE of the RFEH system can be calculated as PCE_sys_ (%), with the general formula given as:

(12) $${\mathrm{PCE}}_{\mathrm{sys}}\;\left(\%\right)=\frac{P_{\mathrm{out},\mathrm{main}}}{P_{\mathrm{in},\mathrm{imn}}}\times 100=\frac{V_{\mathrm{out},\mathrm{main}}^{2}/R_{L}}{P_{\mathrm{in},\mathrm{imn}}}\times 100$$

where P_out,main_ is the output power delivered to the load system, and P_in,imn_ is the input power received at IMN. However, the PCE of the primary path is sufficient to benchmark with the other reported work.

In other words, the system PCE is predominantly determined by the efficiency of IMN (*η*_IMN_), rectifier (*η*_rec_), and PMU (*η*_PMU_).

(13) $$\eta_{\mathrm{System}}=\eta_{\mathrm{IMN}}\times \eta_{Rectifier\;}\times \eta_{\mathrm{PMU}}$$

**Figure 13:**
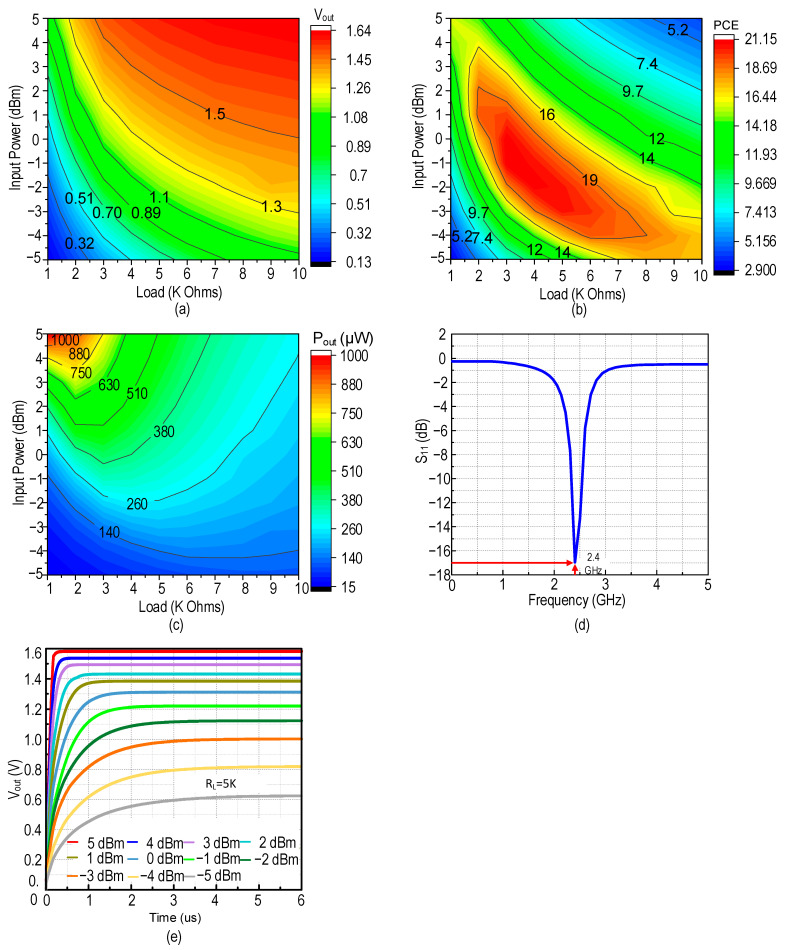
Post-layout Simulation results of RFEH: (**a**) V_out_, (**b**) PCE, (**c**) P_out_, (**d**) reflection coefficient (|S_11_|) versus frequency, and (**e**) transient response.

The input power versus the load in the main path is shown in Figure 13a–e with the corresponding contour plots of V_out,rec_, PCE, P_out,_ and transient response of the RFEH system. Figure 13a shows the contour plot of the primary power path output voltage respective to the load, R_L_. A maximum V_out_ of 1.64 V is observed at P_in_ of 5 dBm and the optimal V_out_ respective to the PCE is shown in the green band. Figure 13b shows the contour plot of PCE, where the proposed system achieves a peak PCE of 21.15% with a 3.3 KΩ load at 0 dBm of input power and obtains a 5 dB wide dynamic range with a PCE of over 16%. Figure 13c observes an output power of 423 µW at peak PCE and a maximum power of 1 mW at 5 dBm of input power. Figure 13d shows the reflection coefficient (|S_11_*|*) which yields an impedance matching at 2.4 GHz with the input power of 0 dBm. At low power, the secondary path produces a sensitivity of −14.1 dBm achieved by the improved voltage conversion efficiency (VCE) of the CP.

The VCE of the CP can be expressed as,

(14) $$\mathrm{VCE}\;\left(\%\right)=\frac{V_{\mathrm{out},\mathrm{cp}\left(\mathrm{actual}\right)}}{V_{\mathrm{out},\mathrm{cp}\left(\mathrm{ideal}\right)}}$$

where V_out,cp(actual)_ is the actual output DC voltage of the CP and V_out,cp(ideal)_ is the ideal output DC voltage of the CP. The CP pumping efficiency is 99.23% at an input voltage of 0.2 V or V_rec_. This shows the proposed CP works well for lower supply voltage, which is preferred in a fully-integrated application. The proposed RVCO achieves a start-up at V_rec_ = 70 mV. The power consumption of RVCO is 953 pW at 0.2-V of input voltage and is well fitted for RF energy harvesting systems.

Table 3 summarizes the performance comparison with state-of-art RFEH systems [14,22,23,24,25,26,27,28,29]. The proposed fully integrated RFEH system has a unique feature that incorporates dual-channel on-chip IMN, rectifier, capacitive DC-DC converters, and PMU. This allows two separate power path operations, one is connected with the appropriate load and the other helps to provide battery assist operation. In addition, the proposed RFEH system has a higher output power of 423 µW at peak PCE, compared to other recent reported work in Table 3 The RFEHs in [22,23,24] have better PCE, but they require off-chip components, which increase the physical form factor to more than 10 times the size of the proposed system, and these bottlenecks in the miniaturization of the device. Further, the RFEH systems [24,25,26,28] require to be fabricated at a cost-ineffective higher process node such as 65 nm/130 nm. In comparison to the on-chip integrated RFEH solutions in [25,26,27,28,29], the proposed work achieves higher sensitivity, PCE, and output power.

## 5. Conclusions

We presented a fully integrated multi-channel RF energy harvesting implemented in 180-nm CMOS. It adopted a shared-auxiliary-biasing technique to obtain *V*_th_ reduction which yields an improved pumping efficiency of 99.23% for the CP circuit. The CP contains a SAB-RVCO, a buffer, and a charge-pump circuit designed and implemented in an integrated dual-input RFEH system. This work proposed a rectifier stacking technique to reduce the impedance and concurrently improve the quality factor Q achieving a 2.4 GHz multi-channel fully integrated RFEH system with 423 µW output power at a peak PCE_system_ of 21.15% and a proposed charge pump support to reach a sensitivity of −14.1 dBm.

## Figures and Tables

**Table 1:** The typical range of the power consumption of various electronic devices [15,16].

Power	1 µW	10 µW	100 µW	1 mW
Device	32 kHz Quarts oscillator	Wearable, Calculator, Passive RFID	Hearing Aid, Sensors [12,13]	Active RFID, Miniature FM receiver, Health monitoring [14]

**Table 2:** The transistor sizing of the proposed fully integrated RFEH system.

Device Parameter		
**Rectifier**	MDP	12 µ
	MDN	6 µ
	C_c_	2 pF
**Charge Pump**	MP	4 µ
	MN	2 µ
	C_p_	5 pF
	BP	2 µ
	BN	1 µ
***R*VCO** Power Consumption @0.2 V is 953 pW	M_P_/M_SP_/M_CP_	12 µ
	M_N_/M_SN_/M_CN_	6 µ
	M_PB_	6 µ
	M_NB_	3 µ

**Table 3:** Performance benchmark with related State-of-Arts.

Reference	This Work	[14]	[22]	[23]	[24]	[25]	[26]	[27]	[28]	[29]
**CMOS** **Technology**	180-nm	130-nm	180-nm	180-nm	65-nm	130-nm	130-nm	180-nm	130-nm	65-nm
**Frequency (GHz)**	2.4	0.9	0.93 & 2.63	0.914, 2.4	2.45	2.4	1.3	3.85	2.4/5.8	0.9
**IMN** **Topology**	LC	LC	LC	Transmission line	LC	TX	TX	TX	LC	TX
**Type of IMN**	On-chip	Off-chip	IPD	Off-chip	Off-chip	On-chip	On-chip	On-chip	On-chip	On-chip
**Additional Technique**	Rectifier stacking, Charge pump	Off-chip Inductor, PCB	Dual band, high-Q IPD passive components,	RCN, PCB	DC-boosted gate	Switch NMOS	Voltage gain, reduce dead zone	-	Bond wire inductor	Step-up stacked transformer
**Rectifier**	3 × 3 + 6 stage CP	5 stage	5 stage, Dickson topology	3 stage	2 stage	5 + 4 stage rectifier	18-stage Half wave rectifier	Dual half-wave rectifier	5 stage Diode based	5 stage
**Sensitivity @1 V**	−14.1 dBm	−12.3 dBm	−16,−15.4 dBm	−16 dBm	−12 dBm	−10 dBm	−11 dBm	−12 dBm	−13 dBm	−16.5 dBm
**Peak PCE**	21.15% @0 dBm ^†^	29.3% @ 0.2 dBm	23.3% @−1 dBm *	43.1&36.5% *	73.6% @ −6 dBm *	15.9% @0 dBm ^†^	0.03% ^†^	1.58% ^†^	14% @0 dBm ^†^	<1%
**Power at Peak PCE**	423 μW ^†^	307 μW *	185 μW *	43 μW *	184.73 μW *	159 μW ^†^	-	-	140 μW ^†^	-
**Size**	1.02 mm^2^	0.17 mm^2^ * (PCB) *	11.6 mm^2^ *	6.1 × 3.24 cm^2^ (FR4) *	1 mm^2^ (PCB) *	2.08 mm^2^	0.05 mm^2^	0.04 mm^2^	1.02 mm^2^	0.28 mm^2^

^†^ On-chip IMN. * Off-chip IMN/non-CMOS.

## Data Availability

Data are contained within the article.
